# Efficacy of acetylcholinesterase inhibitors on reducing hippocampal atrophy rate: a systematic review and meta-analysis

**DOI:** 10.1186/s12883-024-03933-4

**Published:** 2025-02-12

**Authors:** Youssef A. Ismail, Youssef Haitham, Mohammad Walid, Hazim Mohamed, Youssef M. Abd El-Satar

**Affiliations:** 1 Faculty of Medicine, Port Said University, Port Said, Egypt; 2 Faculty of Medicine, Suez Canal University, Ismailia, Egypt; 3 Faculty of Medicine, Arish University, Arish, Egypt; 4 Faculty of Medicine, Helwan University, Cairo, Egypt; 5 Faculty of Medicine, Cairo University, Cairo, Egypt

**Keywords:** Alzheimer’s disease (AD), Mild cognitive impairment (MCI), Hippocampal atrophy, Acetylcholinesterase inhibitors

## Abstract

**Background:**

Neurodegenerative diseases (NDs) are conditions characterized by irreversible progressive degeneration to the nervous tissue and are usually associated with cognitive decline and functional deficits, especially in elderly. Acetylcholinesterase inhibitors (AChEIs) like donepezil, rivastigmine, and galantamine are commonly prescribed to alleviate cognitive symptoms associated with NDs. However, their long-term impact on slowing structural brain degeneration, particularly hippocampal atrophy, remains unclear.

**Objective:**

This systematic review and meta-analysis assess the efficacy of AChEIs in reducing hippocampal atrophy in patients with NDs or clinical syndromes that lead to cognitive decline.

**Methods:**

A systematic search of PubMed, Scopus, Web of Science, and Cochrane databases, since inception till 20th August 2024, identified randomized controlled trials (RCTs) and comparative studies that measured hippocampal volume changes in elderly patients with NDs and other clinical syndromes. Random effect model was employed to estimate the pooled atrophy rates. Subgroup analysis was conducted by disease, dosage, and side of the measurement.

**Results:**

From 5,943 initially screened studies, nine were included in the review, and six were analyzed in the meta-analysis, encompassing a total of 2,179 participants. The meta-analysis showed that donepezil at a 10 mg dose significantly reduced hippocampal atrophy compared to placebo (SMD = 0.44, 95% CI [0.08 to 0.81], *p* = 0.01), whereas the 5 mg dose showed no significant effect on hippocampal volume. Overall, pooled results favored donepezil in reducing hippocampal atrophy (SMD = 0.33, *p* = 0.04), indicating that higher doses are more effective. Among patients with mild cognitive impairment (MCI), both donepezil and vitamin E were associated with a significant reduction in hippocampal atrophy compared to placebo (SMD = 0.27, *p* = 0.01). In contrast, galantamine did not significantly reduce hippocampal atrophy in the overall analysis, but it was associated with reduced whole brain atrophy in APOE ε4 carriers. Further analysis revealed no significant difference in the reduction of right or left hippocampal atrophy in donepezil-treated patients. These findings suggest that donepezil, particularly at higher doses, may have a protective effect against hippocampal atrophy in patients with AD and MCI, while galantamine’s effect may be more limited, especially in certain genetic subgroups.

**Conclusion:**

Higher doses of donepezil (10 mg) significantly reduce hippocampal atrophy in Alzheimer’s disease and mild cognitive impairment, suggesting potential neuroprotective effects. In contrast, lower doses (5 mg) and galantamine showed no significant impact on hippocampal volume, though galantamine reduced whole brain atrophy in APOE ε4 carriers. Dosage and genetic factors are crucial in determining the efficacy of acetylcholinesterase inhibitors in slowing neurodegeneration.

**Supplementary Information:**

The online version contains supplementary material available at 10.1186/s12883-024-03933-4.

## Introduction

Neurodegenerative diseases (NDs) are a type of debilitating conditions characterized by progressive degeneration and irreversible destruction to the nervous tissue. Regardless of the current efforts by medical research to create a medical or surgical solution, the outcome has not been favorable. NDs such as Alzheimer’s disease (AD) continue to be clinically concerning in most older people [1–3]. Neurological disorders and their manifestations are the leading cause of physical and cognitive disability across the globe, currently affecting approximately 15% of the worldwide population [4]. Since the brain regulates many body functions, NDs impact many aspects of human functioning, making it more difficult to perform both basic and complex tasks such as speech and cognitive functions, respectively. While in some cases therapies aim to improve symptoms, relieve pain if it is present, and/or restore movement and balance, most NDs develop without remission [2, 5].

Acetylcholinesterase inhibitors (AChEIs), also known as cholinesterase inhibitors, are a class of indirect parasympathomimetic drugs that act by breaking down the enzyme acetylcholinesterase (AChE), which is involved in the termination of parasympathetic nerve impulses by hydrolyzing the neurotransmitter acetylcholine (ACh) [6]. AChE is involved in various other functions including cell apoptosis, inflammation, morphogenic and adhesive functions as well as oxidative stress that made it a candidate for NDs treatment [7].

Donepezil hydrochloride is a selective reversible AChEI that is often indicated in treatment of all spectrums of AD. Although there is currently no evidence to suggest that donepezil can alter the pathology or the progression of the disease, it has shown efficacy in alleviating specific symptoms by improving cognition and/or behavior in affected individuals [8]. Recent findings suggest that pathophysiology of NDs may be due to neuroinflammation and donepezil has been found downregulating neuroinflammation responses which can be considered modifying of the process of the pathophysiology [8, 9].

Rivastigmine is a carbamate inhibitor that inhibits both AChE and butyrylcholinesterase (BChE). The drug is indicated for use in patients with mild-to-intermediate AD as well as idiopathic Parkinson’s Disease (PD). It has been shown to have potential effects at permanently improving cognitive function as well as slowing down the rate of cognitive decline from NDs [10]. Combination with Donepezil indicated more enhanced cognitive function compared to monotherapy [11].

Galantamine is a selective AChEI and is similar to Rivastigmine in indications and side effects [7]. Galantamine shows high efficacy at improving cognitive function of patients with NDs, particularly those with mild to moderate AD. However, Galantamine trials have shown no evidence of global improvement of the AD patient condition [12].

The hippocampus, which is located in the medial region of the temporal lobe, is a vital part of the brain responsible for learning and memory, as well as spatial awareness and navigation, emotional behavior and regulation of hypothalamic function [13]. Studies showed that tau protein and atrophy in the cerebral cortex often starts in the hippocampus before other parts [14], which leads to the typical early symptoms of AD including memory loss and lowered sensory spatial awareness [15].

Hippocampal atrophy measured by structural magnetic resonance imaging (MRI) is considered one of the best diagnostic and prognostic tools for AD [15]. Patients have been shown to have 10–15% atrophy of hippocampal volume in mild cognitive impairment (MCI), increasing to 15–30% atrophy in cases of early AD [16] and reaching values of up to 50% lower volume in moderate AD [17].

These values show a very strong direct correlation between percentage of hippocampal atrophy and progression of neurodegeneration caused by AD and MCI. The buildup of tau protein has been shown to have direct impact on hippocampal volume, as it is related to necrosis and atrophy of hippocampal tissue [18]. Hippocampal atrophy is also not only a tool to determine diagnosis and prognosis, but is a useful measure to predict the progression of MCI into AD [19], making it a good outcome to measure the efficacy for drugs used to slow down cognitive decline and progression of NDs.

In this systematic review and meta-analysis, we aim to determine and summarize the efficacy of AChEIs at reducing the rate of hippocampal atrophy in elderly patients suffering from NDs.

## Methods

The study was retrospectively registered on PROSPERO and gained approval (CRD42024587839). In preparing this systematic review, we adhered to the guidelines set forth by the Preferred Reporting Items for Systematic Reviews and Meta-Analyses (PRISMA) statement.

### Search strategy and study selection

Guided by the Preferred Reporting Items for Systematic Reviews and Meta-Analysis Statement (PRISMA), we performed a systematic search of PubMed, Scopus, Web of Science, and Cochrane since inception until 20th August 2024 using search strategy (*Supplementary Material*). We screened peer-reviewed full-text articles published in English.

### Data extraction, inclusion, and exclusion criteria

We included randomized controlled trials (RCTs), controlled studies (e.g., cohort studies with control groups), and comparative observational studies (e.g., retrospective or prospective cohort studies). We included any AChEI i.e., (donepezil, rivastigmine, galantamine) with any dosage, admiration routes, and either compared to placebo or not. We only included studies been held on elderly patients with NDs or any clinical syndrome predispose the patient to cognitive impairment and hippocampal atrophy. Our primary outcomes were hippocampal volume (in mm^3^) change measured using MRI from the baseline to the endpoint, and one-side hippocampal volume changes and our secondary outcome was the cognitive enhancement aligned with the atrophy rate. We excluded review articles, letters to the editor, case reports, case series, animal studies, non-peer reviewed studies, conference abstracts, and non-English language studies. We also excluded studies involving healthy participants or patients not diagnosed with pre-existing NDs or any clinical syndrome i.e., MCI.

Three authors applied the selection criteria. The eligibility screening process consisted of two stages. Initially, abstracts were reviewed to determine their suitability. In the subsequent stage, the full-text articles corresponding to the selected abstracts were obtained and further assessed for their inclusion in the systematic review.

A standardized data extraction sheet was used to ensure consistency in capturing information across all included studies. The sheet was piloted on a few studies before full extraction to refine the fields and minimize errors. Two independent reviewers (Mohammed H, Walid M) were responsible for extracting the data from the eligible studies. They worked separately to ensure that bias and errors were minimized. In case of discrepancies, a third reviewer (Abd El-Satar YM) was consulted for resolution.

To verify the quality and consistency of data extraction between the reviewers, interrater reliability was assessed using Cohen’s kappa score. A kappa score of 0.8 indicated strong agreement between the two reviewers, which supported the reliability of the data extraction process.

When necessary, authors of the original studies were contacted to clarify unclear or missing data. Any updates or corrections were incorporated into the final extraction sheet after discussions with all reviewers.

For each paper, detailed information was collected on: study information (author’s name, publication year), sample characteristics (sample size, age, sex, mini mental state examination (MMSE), and Alzheimer’s disease assessment scale-cognitive subscale (ADAS-Cog)), study design, intervention details (description, duration) the control group, the hippocampus outcome measures (total and side changes), and cognitive measure at endpoint (if available).

### Quality assessment

The risk of bias was assessed using the Cochrane Risk of Bias Tool (RoB1) for randomized controlled trials. This tool examines potential biases across several domains, including selection bias, performance bias, detection bias, attrition bias, and reporting bias. Each domain is rated as having a low, high, or unclear risk of bias based on the study design and execution. RoB1 aims to provide a structured and transparent approach for assessing methodological quality, helping ensure more reliable results and conclusions in systematic reviews. Two authors (Ismail YA, Haitham Y) independently assessed the quality of each included study and any disagreement was discussed with and settled by the third arbitrator.

### Statistical analysis

We performed statistical analyses using Microsoft Excel and Review Manager (RevMan), version 5.4 (The Cochrane Collaboration’s guidelines, 2020). Meta-analyses were conducted employing a random effects model in all instances. A *P*-value less than 0.05 was considered statistically significant. For each variable, we reported the mean, and 95% confidence interval (CI). I^2^ is a measure of the heterogeneity of the studies, ranging from 0 to 100%, with greater I^2^ values indicating increased heterogeneity. It is interpreted as follows; values < 40%: may suggest that heterogeneity is not important, while values > 75%: may represent considerable heterogeneity. If heterogeneity was present, the data were pooled using the random effects model. Subgroup analysis was conducted by disease, dosage, and side of the measurement. Sensitivity analysis was also performed to resolve heterogeneity.

## Results

### Study selection

**Fig. 1:**
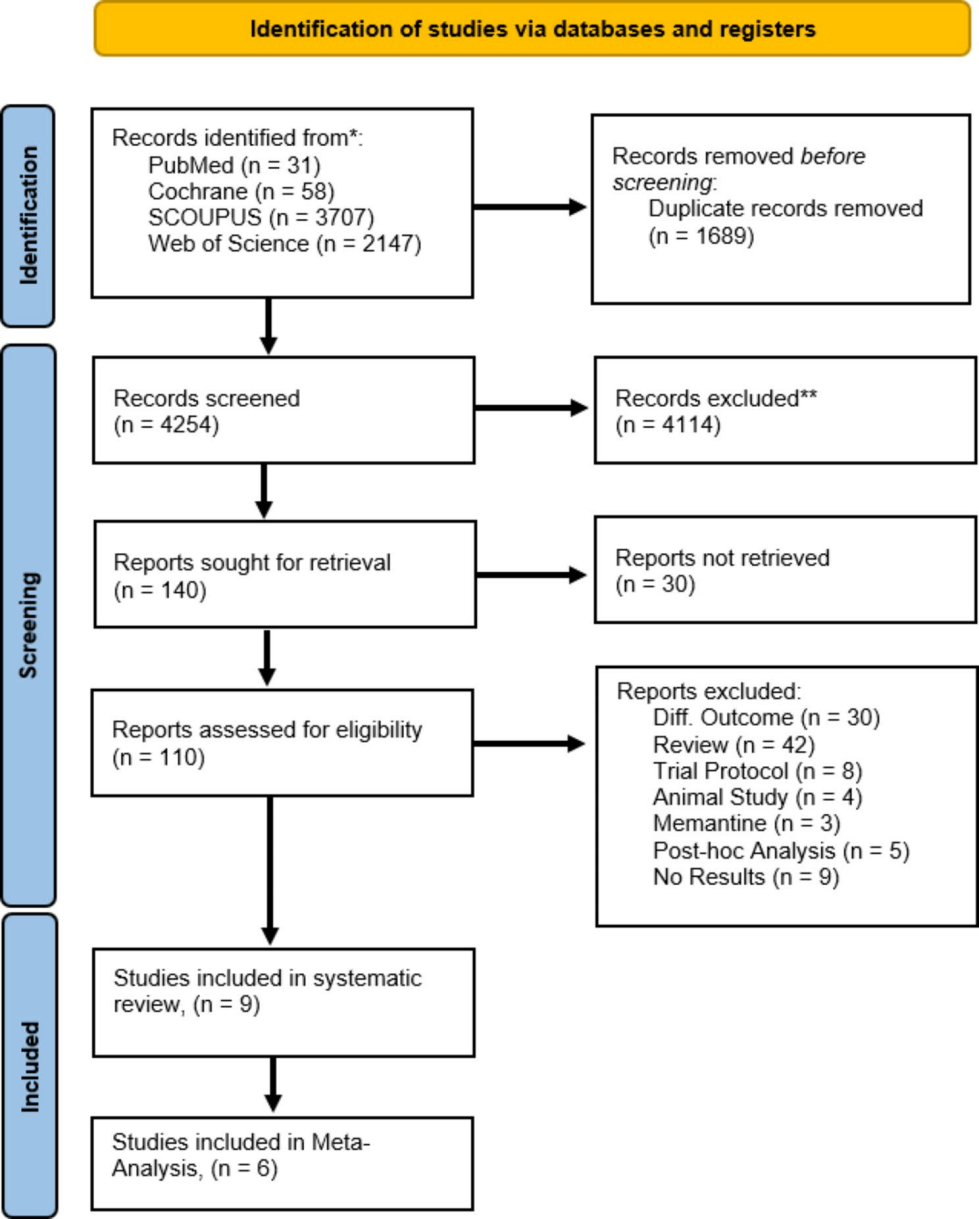
PRISMA flow diagram of the study selection process

In total, 5,943 studies were initially identified. Of these, 5,943 articles were obtained through four electronic database searches: Web of Science (*n* = 2147), Scopus (*n* = 3707), PubMed (*n* = 31), and Cochrane (*n* = 58). After the removal of duplicates and screening of titles and abstracts, 140 studies were assessed for eligibility. Following the assessment against the inclusion criteria, nine full-text articles were included in this systematic review and six were included for meta-analysis (Fig. 1). All these studies were published in the English language in peer-reviewed journals.

### Study characteristics

Summary of the included studies are presented in Table 1. Included studies used a randomized controlled trial study design [20, 22–28] except ***Hashimoto et al.*** [21]. was prospective cohort study. The nine included studies were conducted between 2003 and 2020 across seven countries, including USA (*n* = 3), France (*n* = 1), Japan (*n* = 1), Netherlands (*n* = 1), Canada (*n* = 1), Italy (*n* = 1), and Republic of Korea (*n* = 1). The included studies focused on measuring the effectiveness of AChEIs on total, right, and left hippocampal volume beside the cognitive assessment between baseline and endpoint of each study. Baseline characteristics of the included studies are presented in Table 2. Included studies showed a total of 2179 participants (956 females, 43.8%). The age groups of the participants in the included studies were almost the same, but the baseline MMSE of the included studies varied, five studies with an average baseline of MMSE below 26. The baseline ADAS-Cog of the included studies varied, due to different populations targeted by the studies and multiple modified questionnaires of ADAS-Cog.

**Table 1:** Represents the summary of the included studies

Study ID	Country	Study Design	Study Period	Intervention	Disease	Conclusion
Dubois [20]	France	multicenter double-blind, randomized, placebo controlled, parallel group study	18 months	Patients were randomly assigned to two groups, to either active treatment or placebo [two capsules of 5 mg donepezil (i.e., 10 mg) daily from week 6 to month 12; or placebo.	MCI	Donepezil group exhibited a statistically significant reduced rate of hippocampal atrophy compared with the placebo group There was no significant difference in neuropsychological performance between treatment groups.
Hashimoto [21]	Japan	prospective cohort was compared with a historical control cohort.	12 months	the patients received 3 mg/day of donepezil for 1 or 2 weeks and then 5 mg/day	AD	Donepezil treatment slows the progression of hippocampal atrophy, suggesting a neuroprotective effect of donepezil in Alzheimer’s disease.
Jack [22]	USA	randomized, double-blind, placebo-controlled, parallel-group study	36 months	Participants were randomized into three groups: one received vitamin E, another received donepezil, and the third group received placebos, with all groups also taking a daily multivitamin.	MCI	Results of this study support the feasibility of using MRI as an outcome measure of disease progression in multi center therapeutic trials for MCI.
Krishnan [23]	USA	randomized, double-blind, placebo-controlled, parallel-group study was followed by a 6-week, single-blind placebo washout period.	24 weeks	Patients randomly assigned to the donepezil group received 5 mg/day for the first 28 days and 10 mg/day thereafter.	AD	These preliminary results suggest that donepezil may have a potentially protective effect in Alzheimer’s disease.
Prins [24]	Netherlands	randomized, double-blind, placebo-controlled clinical trial	24 months	Patients were randomized to receive galantamine or placebo for 24 months.	MCI	Patients with MCI who were treated with galantamine demonstrated a higher rate of hippocampal atrophy, compared to placebo group.
Roman [25]	Canada	This investigation was a randomized, double-blind, placebo-controlled,	24 weeks	Participants were randomly assigned 2:1 to donepezil 5 mg or placebo once daily.	Vascular Dementia	Patients treated with donepezil 5 mg/d demonstrated significant improvement in cognitive, but not global, function.
Schuff [26]	USA	Subjects participated in a 3-week single-blind, placebo run-in period followed by a 48-week double-blind period	51 weeks	they were randomly assigned to treatment with 10 mg/day donepezil hydrochloride or placebo	MCI	These findings suggest a treatment effect of donepezil on brain atrophy in aMCI.
Traini [27]	Italy	multicenter, randomized, placebo-controlled, double-blind clinical trial	36 Months	Initially, the protocol plan was to treat patients either with donepezil + choline alphoscerate (treatment group D + CA) or donepezil + placebo (control group D + P) for 24 months	AD	Our findings indicate that the addition of choline alphoscerate to standard treatment with the cholinesterase inhibitor donepezil counters to some extent the loss in volume occurring in some brain areas of AD patients combined with less pronounced cognitive impairment.
Moon [28]	Republic of Korea	-	24 weeks	Donepezil 10 mg	AD	donepezil-treated patients showed significantly increased volumes in the Hip, PCu, fusiform gyrus and caudate nucleus

**Table 2:** Represents the baseline characteristics of the included studies

Study ID	Arms	Number of Patients, *n* (%)	Female, *n* (%)	Age, years Mean (SD)	Baseline MMSE, Mean (SD)	Baseline ADAS-Cog, Mean (SD)
Dubois [20]	Placebo	92	45 (49)	73.7 (6.6)	25.8 (2.6)	12.1 (4.2)
	Donepezil 10 mg	82	44 (54)	73.9 (6.6)	26.2 (2.1)	12.0 (4.3)
Hashimoto [21]	Placebo	93	75 (81)	70.5 (9.1)	21.6 (2.8)	-
	Donepezil 5 mg	54	42 (78)	69.5 (9.5)	21.8 (3.9)	-
Jack [22]	Placebo	54	24 (44.4)	72.0 (7.0)	27.4 (1.9)	11 (4.2)
	Donepezil 10 mg	37	14 (37.8)	72.6 (5.8)	27.4 (1.9)	10.9 (3.8)
	Vitamin E (2000 U/I)	40	16 (40)	73.3 (7.4)	27.9 (1.6)	10.7 (3.6)
Krishnan [23]	Placebo	33	23 (70)	72.4 (10.1)	19.0 (4.6)	-
	Donepezil 10 mg	34	25 (74)	74.4 (7.0)	19.5 (4.8)	-
Prins [24]	Placebo	188	54 (29)	69 (8.6)	-	14 (6.9)
	Galantamine	176	43 (24.4)	68 (9.0)	-	15 (7.2)
Roman [25]	Placebo	326	150 (46)	72.3 (9.028)	23.57 (4.875)	18.64 (10.111)
	Donepezil 5 mg	648	250 (38.5)	73.4 (10.182)	23.49 (5.091)	18.32 (10.182)
Schuff [26]	Placebo	125	54 (43.2)	68.4 (9.9)	27.5 (2.1)	17.8 (7.0)
	Donepezil 10 mg	109	48 (44)	70.6 (9.8)	27.8 (1.8)	17.9 (6.4)
Traini [27]	Donepezil 10 mg	27	12 (46)	74 (6)	20.8 (4)	24.5 (7.3)
	Donepezil 10 mg + Choline Alphoscerate	29	18 (62)	74 (6)	19.8 (2.8)	28 (6.7)
Moon [28]	Placebo	11	6 (54.5)	73 (7.5)	16.9 (5.1)	24.3 (3.4)
	Donepezil 10 mg	11	6 (54.5)	73 (7.5)	18.5 (4.5)	24.6 (6)
	Control	10	7 (70)	70.7 (3.3)	-	-
TOTAL		2179	956 (43.8)			

### Quality of included studies

**Fig. 2:**
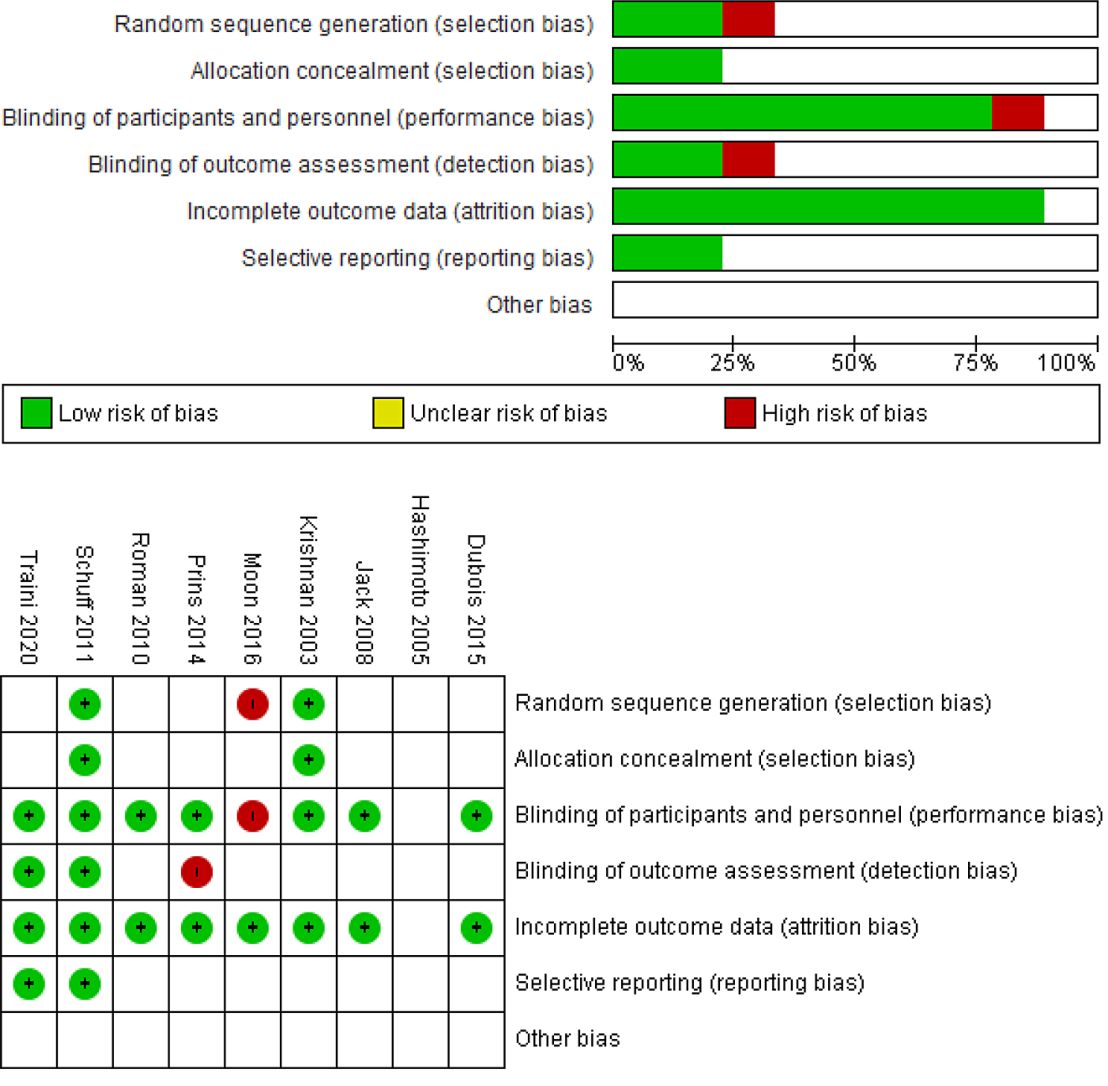
Risk of bias assessment of the included studies (*Hashimoto et al.* is not RCT)

The risk of bias was assessed across various domains for the included studies according to the Cochrane risk of bias assessment tool (Fig. 2). The majority of the studies demonstrated a low risk of bias. However, two studies, ***Moon et al.*** and ***Prins et al.*** were assessed to have a high risk of performance and detection bias due to lack of participant and outcome assessor blinding. Several studies had some unclear or not reported risk of bias in specific domains, but these were not widespread across studies. The quality of the included cohort study was assessed using the Newcastle-Ottawa Scale (NOS). ***Hashimoto et al.***. scored 9 stars, indicating overall good quality.

### Hippocampal volume change

#### Hippocampal atrophy rate reduction between AD and MCI

**Fig. 3:**
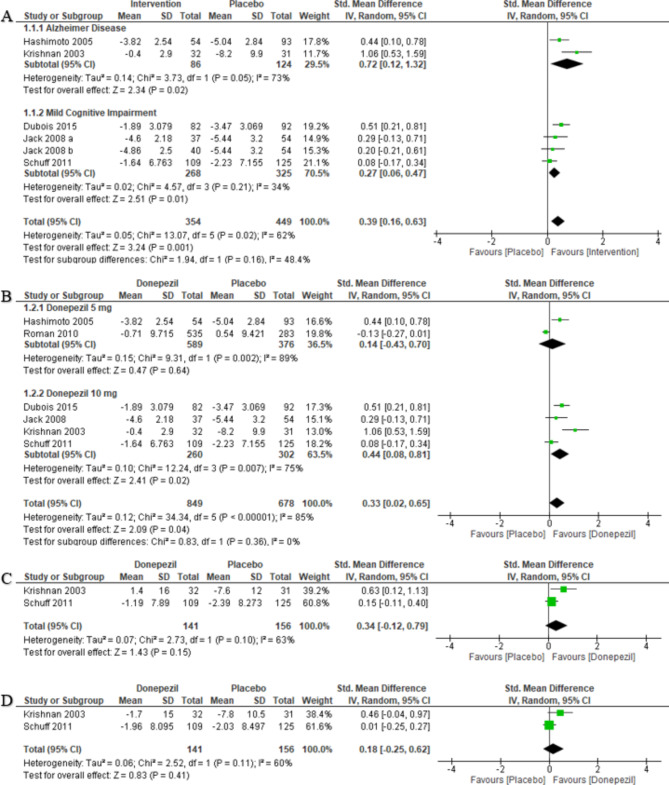
Forest plot of standardized mean difference (SMD) of change in hippocampal atrophy rate; (**A**) subgroup analysis according to the disease; (**B**) subgroup analysis according to dosage of donepezil hydrochloride; (**C**) subgroup analysis of right; (**D**) and left hippocampal sides

The hippocampal atrophy rate reduction between donepezil and placebo in AD patients favored donepezil (SMD = 0.72, 95% CI [0.12 to 1.32], *P* = 0.02). Pooled studies were not homogenous (Chi-square *P* = 0.05, I-square = 73%). The hippocampal atrophy rate reduction between interventions (donepezil and vitamin E) and placebo in MCI patients favored interventions (SMD = 0.27, 95% CI [0.06 to 0.47], *P* = 0.01). Pooled studies were homogenous (Chi-square *P* = 0.21, I-square = 34%). The overall hippocampal atrophy rate reduction between interventions and placebo favored interventions (SMD = 0.39, 95% CI [0.16 to 0.63], *P* = 0.001). Pooled studies were not homogenous (Chi-square *P* = 0.02, I-square = 62%). Two subgroups were not statistically different and with moderate heterogeneity (*P* = 0.16, I-square = 48.4%) (Fig. 3.A). We also conducted pre-specified sensitivity analysis, excluding ***Roman et al.***., as not considered for either of two subgroups criteria (population criteria included to be diagnosed with vascular dementia). In ***Moon et al.***., the rate of hippocampal atrophy reduction in AD patients was statistically significant (*p* < 0.001).

In ***Prins et al.***., the rate of hippocampal atrophy reduction between galantamine and placebo was in favor of placebo, adjusted mean difference = − 0.24 (2.937). Potential differences in patient characteristics, such as gender (*p* = 0.04), and clinical factors like hypertension and ADAS-Cog scores, were significantly different between APOE carriers and non-carriers. Galantamine was associated with a reduced rate of whole brain atrophy, with an adjusted mean difference of 0.17 (0.51), but this effect was observed only in APOE ε4 carriers.

#### Hippocampal atrophy rate reduction with multiple doses of donepezil

The hippocampal atrophy rate reduction between donepezil 5 mg and placebo did not favor either of the two groups (SMD = 0.14, 95% CI [-0.43 to 0.70], *P* = 0.64). Pooled studies were not homogenous (Chi-square *P* = 0.002, I-square = 89%). The hippocampal atrophy rate reduction between donepezil 10 mg and placebo favored donepezil (SMD = 0.44, 95% CI [0.08 to 0.81], *P* = 0.01). Pooled studies were not homogenous (Chi-square *P* = 0.007, I-square = 75%). The overall hippocampal atrophy rate reduction between donepezil and placebo favored donepezil (SMD = 0.33, 95% CI [0.02 to 0.65], *P* = 0.04). Pooled studies were not homogenous (Chi-square *P* < 0.00001, I-square = 85%). Two subgroups were not statistically different and with no heterogeneity (*P* = 0.36, I-square = 0%) (Fig. 3.B).

#### Right and left hippocampal atrophy rate reduction

The right hippocampal atrophy rate reduction between donepezil and placebo did not favor either of the two groups (SMD = 0.34, 95% CI [-0.12 to 0.79], *P* = 0.15). Pooled studies were not homogenous (Chi-square *P* = 0.1, I-square = 63%) (Fig. 3.C). The left hippocampal atrophy rate reduction between donepezil and placebo did not favor either of the two groups (SMD = 0.18, 95% CI [-0.25 to 0.62], *P* = 0.41). Pooled studies were homogenous (Chi-square *P* = 0.11, I-square = 60%) (Fig. 3.D). We also conducted pre-specified sensitivity analysis, excluding ***Dubois et al.***., as considered to be an outlier regarding right and left hippocampal volumes. In ***Traini et al.***., There was a statistically significant difference in favor of Donepezil 10 mg plus Choline Alphoscerate.

### Cognitive assessment between baseline and endpoint of treatment

Only three studies reported the neuropsychological assessment post-treatment. In ***Jack et al.***., and ***Moon et al.***., donepezil-treated groups showed improved neuropsychological symptoms in comparison to placebo groups. In ***Jack et al.***., a statistically significant correlation between rate of hippocampal atrophy, MMSE (*r* = -0.38) and CDR (*r* = -0.49) (*p* < 0.001). In ***Moon et al.***., K-MMSE scores in donepezil-treated patients are positively correlated with grey matter volume of the Hippocampus (*p* = 0.001). In ***Dubois et al.***., There was no significant difference in neuropsychological performance between treatment groups.

## Discussion

The results of this review provided significant insights into the effects of AChEIs on hippocampal atrophy and cognitive outcomes in patients with AD, MCI and vascular dementia.

Annual percentage of change (APC) of total hippocampal volume was the primary outcome. The calculation of the annual percentage changes (APCs) for the MRI efficacy measures was performed according to the following procedure:

$$\eqalign{& {\rm{APC}}\,{\rm{ = }}\,{{{\rm{Value}}\,{\rm{at}}\,{\rm{12}}\,{\rm{month}}\,{\rm{follow}}\,{\rm{up - Value}}\,{\rm{at}}\,{\rm{baseline}}} \over {{\rm{Value}}\,{\rm{at}}\,{\rm{baseline}}}} \cr & \times {{365} \over {{\rm{MRI}}\,{\rm{Interval}}}} \times 100 \cr}$$

Reduction of hippocampal atrophy rate was detected in eight included studies in treatment groups compared to placebo groups, but not reported directly in all studies. Rather, a considerable number of investigations primarily report the outcomes of hippocampal atrophy rate through their period of follow up, aimed at understanding the alterations in hippocampal morphology. All included studies’ results were presented in Table 3.

**Table 3:** Represents the results of the included studies

Study ID	Arms	% of THVC, Mean (SD)	%of RHVC, Mean (SD)	% of LHVC, Mean (SD)	Cognitive Assessment
Dubois [20]	Placebo	-3.47 (3.069)	-3.45 (0.36)	-3.64 (0.39)	No Sig. difference between treatment groups
	Donepezil 10 mg	-1.89 (3.079)	-2.02 (0.39)	-1.81 (0.41)	
Hashimoto [21]	Placebo	-5.04 (2.84)	-	-	-
	Donepezil 5 mg	-3.82 (2.54)	-	-	
Jack [22]	Placebo	-5.44 (3.2)	-	-	Statistically significant (*p* < 0.001) of correlation (spearman r of -0.38 for MMSE and − 0.49 for CDR)
	Donepezil 10 mg	-4.6 (2.18)	-	-	
	Vitamin E (2000 U/I)	-4.86 (2.5)	-	-	-
Krishnan [23]	Placebo	-8.2 (9.9)	-7.6 (12)	-7.8 (10.5)	-
	Donepezil 10 mg	-0.4 (2.9)	1.4 (16)	-1.7 (15)	
Prins [24]	Placebo	-0.24 (2.937)	-	-	-
	Galantamine		-	-	
Roman [25]	Placebo	0.54 (9.421)	-	-	-
	Donepezil 5 mg	-0.71 (9.715)	-	-	
Schuff [26]	Placebo	-2.23 (7.155)	-2.39 (8.273)	-2.03 (8.497)	-
	Donepezil 10 mg	-1.64 (6.763)	-1.19 (7.89)	-1.96 (8.095)	
Traini [27]	Donepezil 10 mg	-	There was a statistically significant difference in favor of Donepezil 10 mg plus Choline Alphoscerate (D + C).		-
	Donepezil 10 mg + Choline Alphoscerate	-			
Moon [28]	Placebo	donepezil-treated patients have reduced rate of hippocampal atrophy (*p* < 0.001)	-	-	K-MMSE scores in donepezil-treated patients are positively correlated with GM volume of the Hippocampus (*p* = 0.001)
	Donepezil 10 mg		-	-	
	Control		-	-	

### Interpretation of findings

#### Hippocampal atrophy rate reduction between AD and MCI

In AD patients, donepezil demonstrated a statistically significant reduction in hippocampal atrophy compared to placebo (SMD = 0.72, 95% CI [0.12 to 1.32], *p* = 0.02), suggesting a substantial benefit for AD patients. The findings for MCI patients were more modest but still indicated a favorable outcome for interventions, including donepezil and vitamin E, over placebo (SMD = 0.27, 95% CI [0.06 to 0.47], *p* = 0.01).

The results indicated that the rate of hippocampal atrophy was significantly reduced in Alzheimer’s patients receiving acetylcholinesterase inhibitors (AChEIs). This finding suggests the efficacy of AChEIs in moderating the progression of cognitive impairment, particularly in patients with mild-to-moderate stages, as reflected by their demographics’ MMSE scores (≤ 21).

In contrast, patients with MCI exhibited higher MMSE scores and a lesser reduction in atrophy rates compared to those with Alzheimer’s disease. Notably, in *Schuff et al.* it was reported higher baseline ADAS-Cog scores for MCI participants compared to other MCI trials, which may have led to less accurate synthesis and demonstration of the data.

Furthermore, it is important to note that the number of patients diagnosed with MCI is approximately three times greater than that of Alzheimer’s patients involved in the analysis.

#### Hippocampal atrophy rate reduction with multiple doses of donepezil

In the reviewed studies, donepezil was administered at two different dosages (5 mg and 10 mg), resulting in varied effects on hippocampal atrophy.

No significant reduction in hippocampal atrophy was observed for the 5 mg dosage compared to placebo, with a pooled SMD of 0.14 (95% CI: -0.43 to 0.70) and a p-value of 0.64, indicating no statistical significance. High heterogeneity (Chi-square *P* = 0.002, I² = 89%) among studies limited confidence in the effectiveness of donepezil 5 mg. This lack of effectiveness may suggest that the lower dose is insufficient to exert a measurable impact on hippocampal atrophy, raising questions about the optimal dosing strategy for this population.

In contrast, donepezil 10 mg showed more favorable outcomes, with a pooled SMD of 0.44 (95% CI: 0.08 to 0.81) and a p-value of 0.01, indicating a statistically significant reduction in hippocampal atrophy, although high heterogeneity (Chi-square *P* = 0.007, I² = 75%) was still present. The significant effect at this higher dosage suggests that increased dosing could be necessary to achieve clinical benefits in reducing hippocampal atrophy, although the variability in response across studies indicates that individual patient factors may play a crucial role in treatment efficacy.

When combining both dosages, the overall effect of donepezil compared to placebo showed a pooled SMD of 0.33 (95% CI: 0.02 to 0.65) and a p-value of 0.04, favoring donepezil for reducing hippocampal atrophy. However, this analysis also exhibited high heterogeneity (Chi-square *P* < 0.00001, I² = 85%). The variability across studies might reflect differences in patient populations, disease severity, or methodological approaches, underscoring the importance of considering these factors in interpreting the results.

Notably, the comparison between the two subgroups (5 mg and 10 mg) revealed no statistically significant difference (*P* = 0.36, I² = 0%), indicating similar effects on hippocampal atrophy without variability between the dosages. This finding raises further questions regarding the dose-response relationship and suggests that the benefits of higher dosages may not be substantially greater than those at lower dosages in terms of hippocampal atrophy reduction. The 5 mg group presented with lower MMSE scores and had a higher proportion of females compared to males, which may influence the results. Notably, two studies included in this group focused on patients with AD and vascular dementia. In contrast, the 10 mg group had higher MMSE scores and a lower proportion of females, with three studies focused on MCI and one study on AD.

#### Cognitive assessment between baseline and endpoint of treatment

Only three studies reported neuropsychological assessments post-treatment, revealing varied outcomes regarding the efficacy of donepezil.

*Jack et al.*. and *Moon et al.*. found that donepezil-treated groups exhibited significant improvements in cognitive symptoms compared to placebo. *Jack et al.*. reported a negative correlation between the rate of hippocampal atrophy and cognitive performance measured by MMSE and CDR (MMSE: *r* = -0.38, CDR: *r* = -0.49, *p* < 0.001). This suggests that increased hippocampal atrophy is associated with cognitive decline. *Moon et al.*. found a positive correlation between K-MMSE scores and grey matter volume in the hippocampus (*p* = 0.001), indicating that cognitive improvement is linked to maintained hippocampal structure.

In contrast, Dubois et al. found no significant differences in neuropsychological performance between treatment groups, raising questions about the consistency of donepezil’s efficacy. This discrepancy may be due to variations in study designs, participant characteristics, or disease severity at baseline.

#### Effect of galantamine on hippocampal and brain atrophy

The included study, by *Prins et al.*, explored the effects of galantamine on hippocampal atrophy compared to a placebo group. Unexpectedly, the placebo group showed a slower rate of hippocampal atrophy, with an adjusted mean difference of − 0.24 favoring the placebo. This finding raises questions about galantamine’s efficacy, potentially due to differences in patient characteristics, such as gender (*p* = 0.04), and clinical factors like hypertension and ADAS-Cog scores, which were significantly different between APOE carriers and non-carriers. Conversely, galantamine was associated with a reduced rate of whole brain atrophy, presenting an adjusted mean difference of 0.17 (0.51), but this effect was only observed in APOE ε4 carriers. These conflicting results underscore the need for further investigation into the underlying mechanisms influencing treatment outcomes.

### Strengths and limitations of the evidence

One of the strengths of this review is the inclusion of RCTs, which provide a higher level of evidence compared to observational studies. Additionally, the inclusion of studies spanning nearly two decades offers a comprehensive view of the field and captures trends in AChEI treatment efficacy over time. The consistency in patient age groups across studies adds to the reliability of the findings, although the heterogeneity in baseline MMSE and ADAS-Cog scores introduces some variability.

However, the review is limited by several factors. First, there was considerable heterogeneity in some pooled analyses, such as the hippocampal atrophy reduction in donepezil-treated patients (I² = 73%), indicating variability in study designs, patient populations, or treatment protocols. This heterogeneity limits the ability to draw firm conclusions across all studies. Additionally, the lack of blinding in two studies increased the risk of performance and detection bias, further weakening the robustness of the findings.

Another limitation is the small number of studies reporting cognitive outcomes. Only three studies provided data on neuropsychological assessments post-treatment, which restricts the ability to correlate hippocampal atrophy reduction with cognitive improvements. This lack of data highlights a gap in the current research and emphasizes the need for future studies to incorporate comprehensive neuropsychological assessments alongside imaging data.

### Implications for practice and future research

The findings of this review reinforce the clinical utility of AChEIs, particularly donepezil, for managing hippocampal atrophy in AD patients. Clinicians may consider AChEIs as part of a comprehensive management strategy for patients diagnosed with AD, with the potential to slow disease progression. For MCI patients, the decision to initiate AChEI treatment should be made cautiously, considering the more modest effect sizes observed.

Future research should aim to address the limitations identified in this review. Specifically, larger RCTs with longer follow-up periods are needed to confirm the long-term effects of AChEIs on hippocampal atrophy and cognitive decline. Additionally, studies should prioritize the inclusion of cognitive assessments to provide a more holistic understanding of the relationship between hippocampal volume changes and functional outcomes.

The heterogeneity in study design and outcomes underscores the need for standardized protocols in future research. This includes uniform criteria for patient inclusion, consistent use of imaging and neuropsychological tools, and well-defined intervention protocols to reduce variability and improve the comparability of results across studies.

## Conclusion

This systematic review provided evidence supporting the use of AChEIs in reducing hippocampal atrophy in AD patients, with some potential benefit in MCI patients.

The comparison between donepezil 5 mg and 10 mg in terms of hippocampal atrophy reduction clearly favors the 10 mg dosage, which showed statistically significant benefits. These findings suggest that the higher dose of donepezil could offer more robust neuroprotection, potentially slowing disease progression in AD patients. However, the substantial heterogeneity across studies indicates that individual patient characteristics, such as baseline cognitive function or disease stage, may influence the degree of benefit from donepezil therapy. Galantamine did not perform well in the study included in the review, with results indicating that it may not effectively reduce hippocampal atrophy, and in fact, the placebo group performed better in terms of slowing hippocampal volume loss. This is a notable contrast to the favorable outcomes seen with donepezil, particularly at the 10 mg dose. As a result, galantamine does not appear to be a superior therapeutic option for slowing hippocampal atrophy based on the findings of this review.

The findings are encouraging but should be interpreted cautiously due to heterogeneity across studies and the limited data on cognitive outcomes. Future research should focus on addressing these limitations to further elucidate the role of AChEIs in slowing neurodegeneration and improving patient outcomes.

## Electronic supplementary material

Below is the link to the electronic supplementary material.

Supplementary Material 1

## Data Availability

All data generated or analyzed during this study are included in this published article.

## Abbreviations

AChEIs: Acetylcholinesterase Inhibitors
AD: Alzheimer’s Disease
ADAS-Cog: Alzheimer’s disease assessment scale-cognitive subscale
APOE e4: Apolipoprotein E epsilon 4 genotype
APC: Annual Percentage of Change
MCI: Mild Cognitive Impairment
MMSE: Mini-Mental Status Examination
MRI: Magnetic Resonance Imaging
NDs: Neurodegenerative Diseases
PRISMA: Preferred Reporting Items for Systematic Reviews and Meta-Analyses
RCTs: Randomized Clinical Trials
