# Supplementary material for: Efficacy of acetylcholinesterase inhibitors on reducing hippocampal atrophy rate: a systematic review and meta-analysis
Source: BMC Neurol. 2025 Feb 12;25:60. doi: 10.1186/s12883-024-03933-4 (PMC11816531; doi:10.1186/s12883-024-03933-4)
Supplement: Supplementary file 1 — Supplementary Material 1 [file 12883_2024_3933_MOESM1_ESM.docx]

**Search formulas of Past Systematic Review;**

Database:

**PubMed**

Date of search:

August 20^th^, 2024

Search strategy:

("cholinesterase inhibitors"[All Fields] OR "cholinesterase inhibitors"[MeSH Terms] OR Acetylcholinesterase inhibitor[Text Word] OR "Donepezil"[Mesh] OR "Rivastigmine"[Mesh] OR "Galantamine"[Mesh] OR "Tacrine"[Mesh]) AND ("Hippocampus"[Mesh] OR (Hippocamp*))

Database

**The Cochrane Library**

Date of search:

August 20^th^, 2024

Search strategy:

#1 MeSH descriptor: [Cholinesterase Inhibitors] explode all trees

#2 "Acetylcholinesterase inhibitor"

#3 MeSH descriptor: [Donepezil]

#4 MeSH descriptor: [Rivastigmine]

#5 MeSH descriptor: [Galantamine]

#6 MeSH descriptor: [Tacrine]

#7 (#1 OR #2 OR #3 OR #4 OR #5 OR #6)

#8 MeSH descriptor: [Hippocampus] explode all trees

#9 (Hippocamp*)

#10 (#8 OR #9)

#11 (#7 AND #10)

Commentary:

The search strategy is an adaptation of the PubMed search strategy.

Database

**SCOPUS, WOS**

Date of search:

August 20^th^, 2024

Search strategy:

((cholinesterase*) OR (anticholinesterase*) OR (Donepezil) OR (Rivastigmine) OR (Galantamine) OR (Tacrine)) AND (Hippocamp*)

Commentary:

The search strategy is an adaptation of the PubMed search strategy.
